# Sleep and Emotional and Behavioral Symptoms in Adolescents with Inflammatory Bowel Disease

**DOI:** 10.1155/2014/379450

**Published:** 2014-05-04

**Authors:** Teija Pirinen, Kaija-Leena Kolho, Merja Ashorn, Eeva T. Aronen

**Affiliations:** ^1^Department of Pediatrics, Children's Hospital, University of Helsinki and Helsinki University Central Hospital, 00029 Helsinki, Finland; ^2^Department of Child Psychiatry, Children's Hospital, University of Helsinki and Helsinki University Central Hospital, Lastenlinnantie 2, 00029 Helsinki, Finland; ^3^Pediatric Research Centre, Tampere University, 33014 Tampere, Finland

## Abstract

The current study assessed the associations between sleep and psychosocial symptoms in 157 Finnish adolescents with inflammatory bowel disease (IBD). Sleep trouble was self-rated in Sleep Self-Report (SSR) and in Youth Self-Report (YSR). Psychosocial symptoms of the adolescents were assessed by the YSR and Child Behavior Checklist (CBCL). Patients reporting sleep trouble had significantly more psychosocial symptoms than their counterparts without sleep trouble. This was shown in the CBCL and YSR scales of total problems (*P* < 0.01), anxious/depressed mood (*P* < 0.05), and aggressive behavior (*P* < 0.01). Additionally, SSR sleep problem subscale scores indicating lower sleep quality (bedtime, sleep behavior) associated significantly with attention problems (*P* < 0.05). These results point out that sleep trouble should be recognized and treated in adolescents with IBD to possibly avoid the emerging of psychosocial symptoms.

## 1. Introduction

Adequate sleep in adolescence is important for healthy development and proper daytime functioning. The evidence suggests that disturbances in the quantity and quality of sleep are associated with emotional and behavioral problems, somatic complaints, problems in academic and social performance, and overall quality of life in adolescents from normal populations [1–4]. Sleep troubles are prevalent in adolescents [5] and even more so in adolescents with somatic problems or diseases such as migraine and tension-type headache [6], juvenile idiopathic arthritis (JIA) [7], irritable bowel syndrome [8], and bronchial asthma [9]. Studies on the effects of low amount or low quality of sleep on the psychosocial well-being of children and adolescents with chronic diseases are limited.

IBD, which includes subtypes of Crohn's disease (CD) and ulcerative colitis (UC), is a chronic relapsing inflammatory condition of the gastrointestinal tract and is most typically diagnosed in late adolescence and early adulthood [10]. Primary causes of IBD or factors underlying the increasing incidence and geographical variation remain obscure [11]. We have earlier reported that sleep disturbances are frequent in adolescents with inflammatory bowel disease (IBD) [12]. The reasons for sleep problems in IBD are so far unclear. Possible causes include, for example, disease associated symptoms (pain, stress, and depression), medical treatment (corticosteroid), and disease-related immune changes [13]. Among adolescents with IBD adequate sleep is especially important since the disease itself and its symptoms, such as uncontrollable bowel function, rectal bleeding, diarrhea, abdominal pain, weight loss, fatigue, and in CD aphthous ulcers and fever, are harsh and potentially cause psychosocial distress and impair daytime functioning [14]. Disturbed sleep may further complicate the disease and burden the adolescent in such a way that behavioral and emotional symptoms may arise. In our earlier report we compared the frequency of sleep problems in adolescents with IBD and population based controls and evaluated the association between the severity of IBD symptoms and sleep problems in IBD patients. We reported that adolescents with active IBD suffered from disturbed sleep more often than those with less active disease or population based controls [12].

The present study aims to extend our previous findings with the same sample by comparing daytime emotional well-being and behavior in IBD patients reporting sleep trouble and patients not reporting sleep trouble. We also studied how sleep quality reported by the adolescents with IBD associated with their emotional and behavioral symptoms. To the best of our knowledge, no earlier reports exist on the associations of psychosocial symptoms and sleep in adolescent patients with IBD.

## 2. Materials and Methods

### 2.1. Participants

Detailed subject recruitment has been described in our recent publication on psychosocial symptoms in adolescents with IBD [15]. Briefly, the postal addresses of 300 patients aged 10–18 years diagnosed with IBD in Finland during the period of 1994−2006, according to the files of the Social Insurance Institution of Finland, were retrieved from the database of the Population Register Center. The files of the Social Insurance Institution include information on all patients entitled to reimbursement for IBD-related medical costs. Postal addresses were available for 287 eligible patients.

Standardized questionnaires (see below) translated into Finnish were sent to the 287 patients and their parents. After one reminder, completed Youth Self-Report (YSR) [16] and Sleep Self-Report (SSR) [17] were received from 160 and 159 adolescents with IBD, respectively. Of their parents, 159 returned Child Behavior Checklist (CBCL) [16]. Of these patients, 52% suffered from UC, 33% from CD, and 12% from unclassified colitis (IC), and 3% did not answer this background question. The mean age of the patients at disease onset was 10 (SD 3.3, range 1–15) years, and the mean duration of the disease at the time of the study was 5.2 years (SD 3.3, range 0.7–13); the mean age of the patient group was 15.4 (SD 2.2) years, and it included 85 boys (53%) [15]. In the present study, 157 adolescents who had filled in the item on sleep trouble in YSR were included, and in assessing sleep quality 156 adolescents were included who had filled in all items in SSR.

### 2.2. Measures of Psychosocial Symptoms

The CBCL and YSR questionnaires include 113/112 problem items, respectively, rated on a three-point scale from 0 to 2 (0 does not apply, 1 applies somewhat, and 2 applies well or often) [16]. The questionnaires were scored using the Achenbach scoring program, which gives empirically based broad-band symptom scales for total problems and internalizing (i.e., emotional) and externalizing (i.e., behavioral) problems [16]. The narrow-band syndrome scales of anxious/depressed, withdrawn/depressed, and somatic complaints are included in the emotional broad-band scale; rule-breaking behavior and aggressive behavior are included in the behavioral broad-band scale. Additionally, the narrow-band syndrome scale reflecting attention problems was utilized here.

*T*-scores for all broad-band and narrow-band scales are reported to ease the valuation of clinical significance of psychosocial symptoms and to enable comparison with other parallel studies [16]. The clinical cut-off point for total, emotional, and behavioral problems broad-band scales is a *T*-score above 63. Scores from 60 to 63 are considered to be in the subclinical range. For narrow-band syndrome scales the clinical and subclinical cut-off points are higher (*T*-score > 70 and *T*-score ranging from 65 to 70, resp.) [16]. In this study, the subclinical cut-off points were used as they are considered more sensitive in samples not representing a psychiatric clinical sample.

### 2.3. Measures of Sleep

The sleep trouble item (item 100 = Trouble sleeping?) in the YSR was used to define whether the patient had (applies somewhat, applies well or often) or did not have (does not apply) sleep trouble. The sleep quality was measured by self-rated SSR questionnaire [17]. The first section of the SSR questionnaire includes three background questions on sleeping (Who in your family sets the rules about when you go to bed? Do you think you have trouble sleeping? Do you like to go to sleep?). Single items in the second section are allocated to three subscales to describe bedtime (12 items, such as Do you fall asleep in about 20 minutes? Do you stay up late when your parents think you are asleep?), sleep behavior (7 items, such as Do you think you sleep too little? Do you wake up at night when your parents think you are asleep?), and daytime tiredness (4 items). Response options are “Often” (5–7 times per week), “Sometimes” (2–4 times per week), and “Seldom or never” (0-1 time per week). Items are rated on a 3-point scale, and for each subscale sum-scores are formulated. In this study, we were mainly interested in measuring correlations between sleep quality (subscales of bedtime and sleep behavior) and psychosocial symptoms and thus the subscale of daytime tiredness was excluded from correlation analysis.

The Ethics Committee of Helsinki University Central Hospital approved the study protocol.

### 2.4. Data Analysis

Statistical analyses were performed using SPSS 17.0 software for Windows. CBCL and YSR items were scored using ADM (version 6.5) scoring software (Achenbach 1999–2006). Mean group differences for total, emotional, and behavioral problems broad-band scales were compared with *t*-test for independent samples (*T*-scores used). The mean group differences for narrow-band syndrome scales were tested with nonparametric Mann-Whitney *U* test (raw scores used). When analysing differences in the frequencies of adolescents with/without sleep trouble scoring over the subclinical cut-off points on the broad-band (*T*-score ≥ 60) and narrow-band scales (*T*-score ≥ 65) in the CBCL and YSR questionnaires, cross tabulation and the nonparametric chi-squared test were used. For correlation analyses we used two-tailed Pearson correlation. Statistical significance was set at *P* ≤ 0.05.

## 3. Results and Discussion

### 3.1. Sleep and Behavioral/Emotional Symptoms as Reported by Adolescents (YSR)

Those 32 (20%) adolescents who reported having trouble sleeping sometimes or often were classified as sleep-troubled. The sleep-troubled patients reported significantly more total problems, anxiety/depression, somatic complaints, and aggressive behavior than the adolescents with no sleep trouble (Table 1). Additionally, adolescents with sleep trouble reported more attention problems than adolescents without sleep trouble, although the difference was only close-to-significant (Table 1).

Nearly half (41%, *n* = 13) of the sleep-troubled adolescents reported a subclinical/clinical number of emotional problems which is significantly more compared to the non-sleep-troubled adolescents (16%, *n* = 20, *P* < 0.01). Of the narrow-band syndrome scales included in the emotional problems scale, sleep-troubled adolescents scored significantly more often over the subclinical cut-off point on the scales of anxious/depressed mood (16%, *n* = 5; non-sleep-troubled adolescents 5%, *n* = 6; *P* < 0.05) and somatic complaints (29%, *n* = 9; non-sleep-troubled adolescents 10%, *n* = 12; *P* < 0.01) while the difference on the scale of withdrawn/depressed was nonsignificant (*P* > 0.05). No group differences between sleep-troubled and non-sleep-troubled patients were found for the frequencies of adolescents scoring over the subclinical cut-off point on the scales of total problems and behavioral problems.

Table 2 shows correlations between sleep problems at bedtime and during the night, as reported on the SSR, and psychosocial symptoms. Broad-band and narrow-band scales of the YSR correlated significantly, though the correlation coefficients were only moderate, with the SSR scale scores indicating sleep quality at bedtime and during the night (SSR subscales: bedtime and sleep behavior) (Table 2).

### 3.2. Sleep and Behavioral/Emotional Symptoms as Reported by Parents (CBCL)

According to the parental reports on IBD patients, adolescents with sleep trouble had more total problems, anxiety/depression, somatic complaints, and aggressive behavior than adolescents not reporting sleep trouble. Here, association between sleep trouble and attention problems was not evident (Table 1).

In parental reports, the *T*-score for total problems scale was above the subclinical limit in 34% (*n* = 11) of adolescents with sleep trouble and in 13% (*n* = 16) of adolescents with no sleep trouble (*P* < 0.01). Significantly more adolescents with sleep trouble had a subclinical/clinical number of emotional problems (59%, *n* = 19) compared with the adolescents without sleep trouble (28%, *n* = 34, *P* < 0.01). Of note, the group difference in the frequency of adolescents scoring over the subclinical limit on the scale of emotional problems was caused mainly by the reports in the scale of somatic complaints. The *T*-score for somatic complaints scale was significantly more often above the subclinical limit in adolescents with (81%, *n* = 26) than without sleep trouble (50%, *n* = 62, *P* < 0.01); the difference was nonsignificant in the syndrome scales of anxious/depressed and withdrawn/depressed and in the behavioral problems scale (*P* = 0.063).

Significant correlations were found for the SSR scales indicating sleep quality (bedtime, sleep behavior) and emotional symptoms (*P* < 0.01), behavioral symptoms (*P* < 0.05), and attention problems (*P* < 0.05) according to the parent reports (Table 2).

## 4. Discussion

This study describes how sleep associates with emotional well-being and behavior among adolescents with a chronic inflammatory disease, IBD. The adolescents with IBD reporting sleep trouble suffered from anxious/depressed mood, somatic complaints, and aggressive behavior more often than those whose sleep was not disturbed. Furthermore, the association between SSR measured low sleep quality and attention problems was evident in these adolescents. The observed prevalence of self-reported sleep trouble (20%) in this patient sample [12] was similar to previous epidemiological reports in normative sample [18].

Community-based studies in adolescents show a robust association between subjective sleep trouble and emotional problems [18–21]. Similarly, in JIA, sleep disturbances seem to be related to anxiety [22]. Consistent with these earlier works, adolescents with IBD, who suffered from self-perceived sleep trouble, seemed to be at a greater risk for anxiety and/or depression. Thus, in clinical work, special attention should be paid to recognize depression/anxiety among those pediatric IBD patients, whose sleep is disturbed. In normative populations, parent-reported sleep problems (parasomnias, enuresis, tiredness, noisy sleep, and insomnia) and objectively measured sleep amount and quality of sleep have significantly related to attention problems and working memory [23, 24]. Also, in the present study SSR measured sleep problems at bedtime/during the night and attention problems were significantly correlated according to both respondents (patients and their parents). Surprisingly, sleep-troubled patients seemed to also have more parent- and self-reported symptoms of aggression. This was not anticipated in this patient group. However, in many earlier studies sleep deprivation or sleep troubles have been associated with aggressive and even violent behavior in normative adolescent samples [25, 26]. It is likely that the low amount/low quality of sleep affects regulatory functions in brains which control emotions and behavior and may thus increase the likelihood of diminished behavioral control [27].

Strength of this study was that the number of patients was high, both the adolescents and their parents served as a source of information, and the psychosocial symptoms of the patients were measured by standardized CBCL/YSR questionnaires. The presence of sleep trouble was defined by a self-rated question of the YSR, which can easily be used as a screening instrument in clinical practice. Additionally, the standardized Sleep Self-Report, SSR, was used here to assess sleep trouble more thoroughly. We have reported earlier that there is some discrepancy in reporting about the presence of sleep trouble by adolescents with IBD and their parents [12]. In the future, sleep among patients with pediatric IBD should be evaluated also with methods such as polysomnography or actigraphy to obtain objective data on sleep in these patients.

## 5. Conclusions

The present results suggest that a further evaluation of psychosocial symptoms is required, especially, among those adolescents with IBD, whose sleep is disturbed according to their own opinion. Sleep troubled adolescents with IBD seem to be at a greater risk for anxious/depressed mood and behavioral and attention problems than those whose sleep is not disturbed. Also, early interventions to improve sleep quality among adolescents with IBD are needed. Due to a cross-sectional study design, conclusions of causal connections here are limited. In the future, longitudinal studies are required to further assess causal connections of subjectively and objectively measured sleep amount/quality and psychosocial symptoms in adolescents with IBD. Also, future studies should find out if improving sleep quality lessens the emotional or behavioral symptoms or if, vice versa, treating the emotional symptoms improves sleep quality in adolescents with pediatric IBD.

## Figures and Tables

**Table 1:** Psychosocial symptoms according to self-report (Youth Self-Report, YSR) and parental report (Child Behavior Checklist, CBCL) in adolescents with inflammatory bowel disease, and with or without self-reported sleep trouble.

	YSR scores according to the presence of adolescent-reported sleep trouble			CBCL scores according to the presence of adolescent-reported sleep trouble		
	YES *n* = 32 (20%)	NO *n *= 125 (80%)	*P *	YES *n* = 32 (20%)	NO *n* = 125 (80%)	*P *
	Mean (SD)	Mean (SD)		Mean (SD)	Mean (SD)	
Total Problems	53.7 (7.4)	47.5 (9.2)	0.001	53.8 (8.7)	49.2 (8.8)	0.008
Emotional Problems	56.1 (9.5)	48.9 (9.5)	<0.001	59.8 (8.8)	54.5 (9.4)	0.005
Anxious/Depressed	55.6 (7.6)	52.6 (4.9)	0.002	54.7 (5.7)	53.0 (5.0)	0.023
Withdrawn/Depressed	57.0 (7.9)	54.6 (6.3)	0.136	56.6 (6.2)	56.5 (7.9)	0.449
Somatic Complaints	60.5 (7.4)	54.9 (5.6)	<0.001	68.3 (8.6)	61.4 (8.2)	<0.001
Behavioral Problems	52.8 (5.9)	49.1 (9.0)	0.030	50.6 (8.0)	47.0 (8.2)	0.030
Rule-Breaking Behavior	53.7 (4.4)	54.2 (5.7)	0.635	52.8 (3.6)	52.8 (4.4)	0.259
Aggressive Behavior	55.0 (5.4)	53.1 (5.0)	0.002	54.4 (4.8)	52.2 (4.6)	0.006
Other						
Attention Problems	54.0 (4.3)	53.4 (4.8)	0.083	53.8 (3.8)	53.2 (4.3)	0.216

*t*-test for broad-band symptom scales of Emotional, Behavioral, and Total Problems. Mann-Whitney *U*-test for narrow-band syndrome scales.

**Table 2:** Pearson's correlation (*r*) between Sleep Self-Report subscale scores indicating sleep quality (Bedtime, Sleep Behavior), and self-reported (Youth Self-Report, YSR) and parent-reported (Child Behavior Checklist, CBCL) psychosocial symptoms.

	Bedtime	Sleep Behavior
	*r*	*r*
YSR		
Total Symptoms	0.48**	0.42**
Emotional Symptoms	0.46**	0.43**
Behavioral Symptoms	0.37**	0.29**
Attention Problems	0.43**	0.29**
CBCL		
Total Symptoms	0.31**	0.25**
Emotional Symptoms	0.25**	0.26**
Behavioral Symptoms	0.21*	0.16*
Attention Problems	0.28**	0.18*

**Significant at level *P* < 0.01;*Significant at level *P* < 0.05.
